# Male-On-Male Child and Adolescent Sexual Abuse in the Caribbean Region of Colombia: A Secondary Analysis of Medico-Legal Reports

**DOI:** 10.3390/ijerph17218248

**Published:** 2020-11-08

**Authors:** Elsa Lucia Escalante-Barrios, Sergi Fàbregues, Julio Meneses, María del Mar García-Vita, Daladier Jabba, Carmen Ricardo-Barreto, Sandra Patricia Ferreira Pérez

**Affiliations:** 1 Department of Education, Universidad del Norte, Km.5 Vía Puerto Colombia, 080001 Barranquilla, Colombia; cricardo@uninorte.edu.co; 2 Department of Psychology and Education, Universitat Oberta de Catalunya, Rambla del Poblenou, 156, 08018 Barcelona, Spain; sfabreguesf@uoc.edu (S.F.); jmenesesn@uoc.edu (J.M.); 3 Department of Education, Universidad de Almería, Carretera Sacramento, Calle San Urbano, s/n, 04120 Almería, Spain; margvita@ual.es; 4 Department of Computer Science, Universidad del Norte, Km.5 Vía Puerto Colombia, 080001 Barranquilla, Colombia; djabba@uninorte.edu.co; 5 Instituto Nacional De Medicina Legal y Ciencias Forenses (Regional Norte), Cra. 23 #5356, 080012 Barranquilla, Colombia; sferreira@medicinalegal.gov.co

**Keywords:** child sexual abuse, sexual assault, male victim, male-on-male, adolescents, children, Colombia

## Abstract

Child and adolescent sexual abuse (CSA) is an important global health problem, especially in non-Western low- and middle-income countries. A number of studies have indicated that, in Latin American countries, male CSA is phenomenon of great concern. However, research on this topic is seriously lacking, and more specifically, on male-on-male CSA. We carried out a qualitative and quantitative secondary analysis of 680 cases of alleged male-on-male CSA that occurred between the years 2017 and 2018 in the Caribbean Region of Colombia. We analyzed the contents of forensic interviews with the alleged victims, conducted by professionals working at the Colombian Institute of Legal Medicine and Forensic Sciences. Our findings indicated a high prevalence of cases of alleged male-on-male CSA among young minors. Most of these cases were allegedly perpetrated by offenders known to the victim and involved high levels of violence. Evidence-based and culturally grounded preventative actions, such as training-based programs for teachers and parents among other public health initiatives are needed to address this type of CSA. Further research is also required to gain a more fine-grained understanding of the cultural and social context of CSA in the Caribbean Latin American countries.

## 1. Introduction

Numerous studies provide strong evidence for considering child and adolescent sexual abuse (CSA) an important global health problem. These epidemiological studies have investigated CSA in Western high-income countries and in non-Western low- and middle-income countries [1,2,3,4]. Furthermore, a large body of literature has reported the short-term and long-term consequences of CSA on both the physical and mental health of the victims [5,6,7,8]. These consequences include anal injury and sexually transmitted infections [9], anxiety and depression [10], post-traumatic stress disorder [11], attention deficit hyperactivity disorder [12], and the internalizing and externalizing of behavior problems [13].

The prevalence and severity of CSA is even greater in non-Western low- and middle-income countries due to the high incidence of several risk factors, such as violence, poverty, disintegration of family structures, and social strain, as reported by Al-Mahroos [14], Oyemade [15], and Ross et al. [16]. According to UNICEF statistics, in Latin American countries, CSA is a major threat, as evidenced by the 228 children that are sexually abused hourly [17]. In Colombia, a recent national survey on violence against children and youth revealed that more than one-quarter of children and adolescents between 13 and 17 years had experienced some type of sexual violence within the previous year [18]. As argued by Quintero-Aguado et al. [19], this high prevalence of sexual violence in Colombia could be the result of a cluster of risk factors for CSA that are common in this country, including intrafamilial violence, lack of family cohesion, poor living conditions, low socioeconomic status of the population, low literacy, individual and social stress, drug addiction, and a lack of child protection due to armed conflict.

While the literature on CSA frequently features males in the role of offenders, ample evidence shows that males can also be victims of sexual assault. In 2011, a global meta-analysis of the prevalence of CSA showed that the average prevalence of sexual violence against girls was 18%, while the average prevalence of sexual violence against boys was 7.6% [20]. Likewise, an international review of 55 studies carried out in 24 countries revealed that the estimated prevalence of CSA ranges from 8% to 31% in girls, and from 3% to 17% in boys [21]. While on a world-wide scale, girls are more prone to sexual abuse, a systematic review by Thornton and Veenema [22] confirmed important differences in the prevalence of CSA by gender between Western high-income countries as compared to non-Western low- and middle-income countries, with higher prevalence among males in the latter in certain cases. Although the accuracy of data on this topic may be questioned on account of high levels of underreporting [23], the review cited above showed that in countries such as Brazil [24], Mexico [25], and Nicaragua [26], boys tended to experience a prevalence of CSA that was equal to or greater than that of girls. This trend was confirmed in the previously cited Colombian survey, which reported a slightly higher percentage of males (28.8%) versus females (27.3%) that had recently experienced sexual violence [18].

Although published evidence indicates a high frequency of male children and boys experiencing sexual assaults, research on male CSA is scarce, especially compared to the higher number of studies carried out with female victims [5,22]. Most of the studies addressing the topic of male CSA have been based on mixed-gender samples in order to compare the prevalence of CSA between the two genders. In contrast, only a small number of studies have addressed more detailed aspects of CSA among males, such as the characteristics of the victims, the circumstances of the assaults, and the type of sexual abuse. In a review that analyzed the global prevalence of CSA among children under 18, Barth et al. [21] identified 55 studies, of which only four were based on male-only samples. Similarly, Veenema et al. [23] reviewed 44 publications investigating CSA in non-industrialized countries and found that only four included victims who were exclusively male. The underrepresentation of male CSA in the academic literature can also be found in policy and institutional documents. A recent European Parliament report on sexual violence against minors in Latin America included only the forms of violence experienced by girls, while ignoring those experienced by boys [27].

In addition, of the few existing studies addressing CSA among males, none, to our knowledge, has focused exclusively on male-on-male sexual abuse, that is, the type of sexual assault perpetrated by men against male minors. The scarcity of empirical knowledge on the prevalence and characteristics of male-on-male sexual abuse among minors is consistent with the small number of studies examining this type of sexual assaults in adult populations, as reported in a recent review by Ionnaou, Hammond, and Machin [28]. More research on male-on-male CSA is needed since, as stated in a recent RAND corporation report, “research on this type of sexual assault, however, is still mostly exploratory in nature, and we still do not have a complete picture of its prevalence, the characteristics of these types of perpetrators, or the circumstances surrounding these assaults” [29].

The lack of research on male CSA, and more specifically, on male-on-male CSA, can be partially explained by the under-reporting of this type of abuse due to sociocultural attitudes such as social stigma attached to abuse, potential humiliation, and threats to families [23,28,30,31]. This lack of reporting is even greater in middle-income countries, such as Colombia, where homophobia tends to be highly prevalent due to the cultural ideals of ‘machismo’ and hegemonic masculinity [32]. In this country, core cultural values, such as religiosity, traditional morals, and familialism, are key determinants of negative attitudes towards the lesbian, gay, bisexual, transgender, queer/questioning, and intersex (LGBTQI) communities [33]. These attitudes frequently discourage children from reporting incidents of CSA to authorities, and in some cases parents prevent their children from contacting the authorities [23]. This unwillingness of parents to report sexual assaults may partly explain not only the scarcity of empirical research on CSA in Latin American countries [23,34], but also the low number of studies of CSA, and particularly of male CSA, based on samples of children under 10 years of age, as reported in the review by Veenema et al. [23].

To fill this gap in the literature, the purpose of this qualitative and quantitative secondary analysis study was to examine the characteristics and context of alleged sexual abuse of male minors perpetrated by males in the Caribbean region of Colombia. The following three objectives were addressed: (Objective 1) to describe the characteristics of the alleged victims and to describe the details of the alleged assaults of male-on-male CSA in the Caribbean region of Colombia between the years 2017 and 2018; (Objective 2); to qualitatively describe the alleged victims’ narratives of the sexual acts they reported having been subjected to or forced to perform; (Objective 3) to quantitatively analyze the relationship between the types of sexual acts and the characteristics of the alleged victims as well as the details of the alleged sexual assaults.

## 2. Materials and Methods

### 2.1. Study Design

We carried out a secondary analysis of a medico-legal dataset of case records of alleged CSA during the years 2017 and 2018 in the Caribbean region of Colombia, provided to us by the Colombian Institute of Legal Medicine and Forensic (CILMFS). Following Boslaugh’s [35] definition of secondary data analysis, we analyzed information that was originally collected by professionals working at the CILMFS (rather than by our research team) for purposes other than those of the current study. Previous studies in the field have also conducted secondary analyses of archived CSA national data [36,37,38,39]. Several authors have argued that differences in victim age cut-off points in epidemiological research studies relating to CSA are likely to result in findings that are not directly comparable [40]. In line with several previous studies in the field [3,41], we used 18 years as the age cut-off point (this is the age of majority in Colombia). In accordance with the practice of the CILMFS, we adopted the World Health Organization’s (WHO) definition of CSA, stated as “the involvement of a child in sexual activity that he or she does not fully comprehend, is unable to give informed consent to, or for which the child is not developmentally prepared and cannot give consent, or that violate the laws or social taboos of society” [42].

### 2.2. Description of the Dataset

The dataset included all the cases of CSA reported in the years 2017 and 2018 to the CILMFS in the Departments of Atlántico, Bolívar, Magdalena, Sucre, Guajira, and San Andrés. The initial population in the dataset consisted of 6113 cases of CSA, aged between 0 and 17 years at the time of the reported sexual assault, and allegedly perpetrated by both sexes. Of these, we selected 680 cases involving male minors and allegedly perpetrated by males. Each case recorded in the dataset included descriptive information on the alleged victims (i.e., gender, age, educational level, and town of residence) and the alleged assaults (i.e., gender of the alleged offender, day of the week when the assault took place, location), and textual data deriving from the verbatim transcript of a forensic interview on the alleged sexual assault on a particular minor. According to documentation made available by the CILMFS [43], the forensic interviews followed the child interview model described by Faller [44], which involves a medical examination of the minor along with a single interview. This model has proven to be effective in a large number of studies examining allegations of CSA [44,45,46].

To avoid unnecessary repetition and possible distortion of data, as well as trauma to the minor being examined and interviewed, the professionals involved coordinated the medical examination and the interview questioning. Data collection procedures followed the WHO guidelines [42] and the provisions of Colombian Law for attention to alleged victims of sexual violence. The provisions of both institutions emphasize the importance of respecting the dignity and fundamental rights of the minor. The interviews were carried out privately in a quiet room by professionals working at the CILMFS. In accordance with typical practice in the child interview model [44], the minors were accompanied by an acquaintance or family member, who helped to manage the minor’s initial anxiety and provided additional information on the background of the alleged assault. Subsequently, the interview was carried out with the minor alone, if he agreed. Otherwise, the accompanying adult remained in the room outside the line of sight of the minor and did not participate in the interview. Only the one or two adults and the one minor were in the room.

Following McDermott-Steinmetz-Lane [47], the interview was done in three phases. The initial rapport building phase aimed to tranquilize the minor, while the professional evaluated his verbal fluency and ability to relate prior experiences using the following three types of questions: (1) general questions in a neutral tone inquiring about, for example, the minor’s name, age, and birthdate; (2) questions about the minor’s family, school, and social environment; and (3) questions aimed at determining the minor’s neurodevelopment (e.g., whether he counts using one or two hands and whether he can name the parts of his body). The second, information-gathering phase, aimed to establish the context of the alleged incident and the history of the alleged victim, while exploring the minor’s emotional, cognitive, and physical state. The interviews were structured to obtain a spontaneous chronological and sequential narrative of the reported incident. Open-ended questions were initially used preferentially to elicit lengthier and more detailed accounts of the incident. Several authors [48,49,50] have noted that open-ended questions are more likely to elicit more coherent, reliable and accurate narratives of incidents involving CSA. When interviewers needed to focus the minor’s attention on details of the incident, they used option-posing utterances with a “something else” option [51] as follow-up questions to ask the minor to affirm, negate, or select an option without implying that a particular response was expected (e.g., “Where did the incident occur? In the living room? In the bedroom? Or somewhere else?”). To facilitate communication, typical prompts were used, such as “tell me more” or “you said that…”. Directive questions and presumptive utterances of any sort were avoided. In the third and final closure phase, the interviewer emphasized that the minor should feel no personal guilt about the incident and can count the aid of the accompanying adult as a protecting presence. In accordance with the established guidelines of the WHO [42], the interview was stopped whenever the minor showed signs of discomfort or an inability to coherently relate his experience in order to avoid re-victimization or traumatization as a result of unnecessarily repetitive questioning. In the dataset, a total of 44 interviews were halted for these reasons.

### 2.3. Coding and Analysis

We performed a qualitative and quantitative analysis of the textual data derived from the verbatim transcripts of the forensic interviews relating to the 680 cases of alleged male-on-male CSA. The initial analysis was qualitative, and the second analysis was quantitative. For the qualitative part, a qualitative descriptive approach [52] was followed to accurately describe the narratives of the alleged victims with regard to the types of sexual abuse that they allegedly experienced. Since qualitative description involves a low level of interpretation of the studied events, it is particularly appropriate when, as in the present study, researchers are interested in capturing the participants’ voices and experiences rather than the researchers’ perspective [53]. In this study, using qualitative description allowed us to stay close to the data and to develop a nuanced, in-depth, and contextualized portrait of the types of sexual acts described by the minors. The qualitative analysis followed the eight steps of qualitative content analysis described by Schreier [54]. In steps 1 and 2, we formulated the objectives and selected the material for the analysis (i.e., medico-legal reports). In step 3, we developed a coding frame which included categories representing the following details of each alleged assault: the day of the week when it occurred, the place, the relationship with the alleged offender, whether money was offered, and the type of sexual act the minor reported having been subjected to or forced to perform. For the latter category, we used a classification of sexual acts based on the previously cited systematic review of the literature on male-on-male sexual assaults by Ionnaou et al. [28]. In steps 4 and 5, we piloted the coding frame by carrying out an intercoder agreement test with a random sample of 20% of the case reports. During this process, two researchers (E.E. and S.F.) independently coded the case reports, achieving a high degree of agreement as indicated by Kappa values ranging from 0.73 to 0.97. As a result of the piloting, in step 6, we made slight modifications in the codebook, specifically in some of the categories associated with the type of sexual act. Step 7, which comprised the main analysis phase, involved the same two researchers (E.E. and S.F.) each coding fifty per cent of the remaining case transcripts. During this process, the two researchers were in constant communication and negotiated agreement on any cases that gave rise to doubt. Finally, in step 8, we interpreted and presented the findings in tables, side-by-side with exemplar quotes. Following the qualitative phase of analysis, we carried a quantitative analysis of the frequency of occurrence of each qualitative code. Chi-square tests were used to analyze associations between the types of sexual acts reported and the characteristics of the alleged victims, as well as the details of the alleged sexual assaults.

### 2.4. Ethics

All the data used in this study had been previously anonymized by professionals working at the CILMFS. The study procedures were approved by the Institutional Review Board of the Universidad del Norte (Acta n°180). Before the medical examination and interview, written informed consent was obtained from the parents of the minor or accompanying adults.

## 3. Results

### 3.1. Characteristics of the Alleged Victims and Details of the Alleged Sexual Assaults (Objective 1)

As shown in Table 1, the mean age of the minors was eight years. The majority of the minors (55.6%) had completed preschool education, 25.7% had completed primary school, 14.9% had no formal education, and only 3.2% had completed secondary school. In order to assess poverty, we used the Unsatisfied Needs Index (UNI) (“Necesidades Básicas Insatisfechas”) of the municipality of residence of the minor. Based on census data from the Government of Colombia [55], the UNI is a proxy measure of the level of social and economic development of each municipality, as it quantifies the percentage of population with unmet needs (i.e., below an established threshold) in areas such as quality of housing, crowding, income and education of the household head, and school attendance of minors. Ranging from 0 to 100, the UNI values were divided into quartiles, with quartile 1 indicating a lower percentage of municipalities with unmet needs and quartile 4 indicating a higher percentage. Table 1 shows that almost three-quarters of the minors (73.5%) lived in municipalities in quartile 1, 20.2% lived in municipalities in quartile 2, and only 3.8% and 2.5% lived in municipalities in quartiles 3 and 4, respectively.

Table 2 shows that more than three-quarters (77.4%) of the alleged assaults occurred on a weekday, while 22.6% happened during the weekend. The alleged assaults took place at the minor’s home in 31.9% of the cases, at the alleged offender’s home in 29.6% of the cases, in a public place (e.g., outdoors, park) in 18.1% of the cases, and in other locations in 20.4% of the cases. The cases in which both the minor and the alleged offender lived in the same home have been included in the category ‘minor’s home’. Seventy-two per cent of the alleged assaults (72.1%) occurred in municipalities in quartile 1 of the UNI, 21.3% in municipalities in quartile 2, and 4.1% and 2.5% in municipalities in quartiles 3 and 4, respectively. The most frequent alleged offenders were third-degree relatives, such as cousins and uncles (27.1%), neighbors (19%), other acquaintances (16.6%), and first- and second-degree relatives, including fathers, stepfathers, brothers, stepbrothers, grandfathers, and step grandfathers (14.9%). Other types of alleged offenders included friends of the minors (8.5%), strangers (8.2%), family acquaintances (3.5%), and teachers (2.2%). Finally, in sixty-eight per cent of the alleged assaults (10%), the alleged offender reportedly offered money for sex.

### 3.2. Alleged Victim’s Narratives of the Sexual Acts (Objective 2)

In this section we describe the narratives of the sexual acts provided by the alleged victims -including a few comments from the accompanying adults—which we identified using qualitative content analysis. We identified several types of sexual acts, which we then organized according to the typology proposed by Ionnaou et al. [28]. These authors classified in two types the sexual acts occurring in male-on-male assaults: acts the minor was subjected to (i.e., those in which the minor reported to having had a more passive role), and acts the minor was forced to perform (i.e., those in which the minor reported to having had a more active role). All the acts included in this typology were present in our data, including a few additional acts that were not considered by these authors, such as kissing the minor’s body, watching pornography, exhibitionism, and taking and sending nude photos. Most of the minors reported having been subjected to two or more types of acts in the same assault. Therefore, the numbers of cases are not mutually exclusive. For each type of act, when the information was available, we identified additional details, such as the following: grooming behaviors or strategies used by the alleged offenders, severity of the sexual acts, context of the alleged assault, and any preceding related sexual acts.

#### 3.2.1. Sexual Acts the Minor Reported Having Been Subjected to

*Anal penetration (penis)*: In 47.1% of cases (*n* = 320), the minor reported having been anally penetrated by the alleged offender. Minors usually stated that the offenders threatened them to prevent them from reporting the assault (“*he said that if I told my mother, he was going to beat me*”, minor, 10 years old) and that, during the assault, the offender used physical violence, such as beating him (“*he grabbed by the neck and hit me*”, minor, four years old), physically restraining him (“*he covered my mouth*”, minor, five years old) and forcefully removing clothing (“*he took my pants down to do it to me*”, minor, 13 years old). Minors also reported having felt pain during the assault and afterwards. In fact, especially among young children, the parents or other family members reported in the interview that they had discovered that the minor had been anally penetrated when they observed unusual behavior such as not wanting to speak, crying, or complaints of soreness (“*he said to me*: ‘*mom, my behind hurts*’”, mother of the minor, five years old). Having noticed these symptoms, the family members explained that they had physically examined the minor and noticed evidence of aggression such as red marks on their skin or inflammation, blood in the stool, blood stained clothing, or semen in the anus (“*his penis was scratched and there was a blob of liquid on his buttocks*”, mother of the minor, four years old).

*Fondling/touching*: In 41.2% of cases (*n* = 280), the minor reported that the alleged offender had touched or stroked the intimate parts of the minor with his hands or penis. Minors noted that this act usually occurred as a prelude to other acts, such as anal sex, fellatio, or masturbation, although it sometimes also occurred alone. In such cases, the alleged offender may have decided voluntarily to not continue with the assault (“*the offender touched my penis through my clothes, but didn’t do anything else*”, minor, six years old) or else stopped the aggression because of the resistance of the minor (“*he started touching me down there, through my clothes, and I slapped him*”, minor, seven years old). A common pattern in this type of cases was that the reported assault was not a one-time incident but rather it occurred repeatedly whenever there was an opportunity (“*a cousin, when we were alone (…) he rubbed me all over my body*”, minor, six years old).

*Anal penetration (digital or object)*: In 9.4% of cases (*n* = 64), the minor reported that the alleged offender had penetrated the minor either with his fingers or with other objects, such as sticks, toys, or tools. Minors stated that this type of act tended to produce physical injuries and pain (e.g., anal complaints such as fissures, pain, bleeding, dilatation) (“*my stepfather stuck his finger here [he indicates his buttocks], I bled on the bed and he beat me, and it hurt*”, minor, six years old). Minors also noted that alleged offenders sometimes threatened or bribed them to keep the incident secret (“*he sells mangos and he asked to take off my clothes and he put his finger in my anus; he told me he would do it every day or else he was going to kill me*”, minor, 10 years old).

*Fellatio/Oral intercourse*: In 7.8% of cases (*n* = 55), the minor reported that the alleged offender performed oral sex on him. Minors stated that the offender persuaded them to be subjected to different types of sexual molestation, ranging from oral genital contact to fellatio (“*he touched my penis with his mouth (…) he said that he was going to give me a present*”, minor, four years old). Younger minors mentioned that oral intercourse may have happened during daily routines such as taking a shower (“*my uncle was giving me a bath and he did bad things to me (…) he sucked my penis*”, minor, five years old) or doing recreational activities, such as watching television. Older children and adolescents frequently identified homosexuality as a known characteristic of the alleged offenders (“*I walked out of my house and a homosexual from Sabanalarga, I don’t know his name, said he would give some money and begin to suck my penis*”, minor, 14 years old). Minors also reported fear of catching a disease (“*I became afraid that I might get a disease*”, minor, 14 years old).

*Masturbation*: In 7.2% of cases (*n* = 53), the minor reported having been subjected to acts associated with masturbation, including masturbation of the minor by the alleged offender and masturbation by the alleged offender in front of the minor. In some cases, the act was finished with an ejaculation by the minor (“*he once masturbated me until I ejaculated*”, minor, 14 years old). Minors frequently reported that this type of act had been perpetrated multiple times by the alleged offender (“*when I was five, my uncle touched my penis with his hands, and masturbated me (…) he did it twice a week*”, minor, 13 years old). Masturbation may be the starting point for alleged offenders that go on to perpetrate other sexual acts (“*at the beginning I had to masturbate him (…) after a while, it was not just this, he also wanted oral and anal sex, and I did it several times*”, minor, 15 years old). Some minors reported that the alleged offenders employed strategies for building victims’ trust, gaining compliance, and maintaining the victims’ silence (“*he told me to trust him and to give my soul to god*”, minor, 14 years old).

*Kissing the minor’s body*: In 6.0% of cases (*n* = 41), the minor reported that the alleged offender kissed the body of the minor. Minors noted that, without consent, the alleged offender had kissed parts of their body, such as the cheeks, the neck or the buttocks, and that sometimes this act preceded other sexual acts, such as anal penetration, oral intercourse and masturbation (“*he was kissing me all over, on the mouth, the neck, the buttocks, the penis, everywhere, then he put his finger in my anus, then he put his penis in my anus*”, minor, nine years old).

*Watching pornography*: In 4.3% of cases (*n* = 29), minors reported having been subjected to watching pornography. Minors reported that alleged offenders initiated the sexual encounter by showing pornographic videos, and then they were subjected to anal penetration, oral intercourse, or other sexual acts (“*my cousin took the cellphone and he put on videos of naked women (…) he asked to lower my pants and he put his penis in my anus*”, minor seven years old). After watching pornography with minors, alleged offenders sometimes satisfied their needs for other types of sexual acts by using threats, violence or fear (“*he started to show me pornographic videos and he said ‘see how my penis is getting hard and you have to suck it because otherwise I will hurt your mother or grandmother’; he grabbed me by the hair and forced me to do it*”, minor, 15 years old). Minors reported that, in this type of sexual act, alleged offenders used several grooming methods, including seduction or using enticements such as offering candy, money or other gifts (“*he made watch homosexual pornography on my home computer, and he always had an excuse to stay in my house, or he brought a PlayStation that he has*”, minor, 13 years old).

*Exhibitionism*: In 4.1% of cases (*n* = 28), the minor reported that the alleged offender exposed his genitals to the minor. When this type of act occurred, the alleged offenders often fondled or touched the minor, or requested other types of sexual favors. While the alleged offenders did not use weapons to commit the sexual assault, in some cases they physically threatened or manipulated the minors (“*he told me not to tell my mother that he was showing me his penis because otherwise he would keep my mother in prison or I could lose my family*”, minor 13 years old).

*Alleged offender taking or sending photos*: In 1.8% of cases (*n* = 12), the minor reported that the alleged offender either took photos of the minor’s nude body or sent sexual photos to him. Young children in particular stated that the offender used the Internet, especially Facebook, to send unwanted sexual photos, particularly photos of the offender’s penis (“*he wrote that he wanted to meet me, he sent me a picture of his penis*”, minor, 13 years old). Minors also stated that the alleged offenders sometimes used these photos to send an unwanted request to do something sexual. Sending sexual photos seemed to be a trigger used by the alleged offender to contact the minor. Once photos were sent, the alleged offender used them to convince the minor to set up a face-to-face meeting (“*through Facebook, he asked me to go to his home, he was sending me photos of his penis*”, minor, 14 years old). Narratives revealed that offenders took and kept pictures of the minors to satisfy their sexual needs (“*when the baby was about two months old, his father used to take off his diaper and rubbed his penis until it got hard and took photos. I thought this was normal and he had the photos of the baby and his penis on the cellphone*”, mother of the minor, one year old).

#### 3.2.2. Sexual Acts the Minor Reported Having Been Forced to Perform

*Fellatio/oral intercourse*: In 14.6% of cases (*n* = 99), the minor reported that the alleged offender forced the minor to perform oral sex on him. Minors reported that this type of sexual act was usually accompanied by other types of acts, such as touching, anal penetration, masturbation and oral intercourse (“*he came by the house when my mother wasn’t home and he asked me to go to the bedroom, and I went. Then, he asked me to do things like sucking his penis and kissing his chest*”, minor, 13 years old). In the cases when this type of act was described as a one-time incident, the minor often reported a higher level of physical violence and restraint (“*he hit me in the stomach*”, minor, 15 years old). Minors described the use of tactics such as threats and intimidation by the alleged offender to maintain the secrecy of the act (“*he told me not to tell my mother and if I said anything to her, he was going to get mad and hit me. Because I was afraid, I didn’t say anything*”, minor, nine years old).

*Anal penetration (penis)*: In 4.9% of cases (*n* = 33), the minor reported that the alleged offender forced the minor to anally penetrate him. Minors noted that they were frequently offered money by the offender (“*one day I was on the mountain with Johan killing birds, then Orlando came and he grabbed me here [he indicates his legs] and asked us to penetrate him anally and that he was going to give two thousand pesos*”, minor, eight years old). Minors also said that sometimes this type of act was carried out in groups (“*one day Juan called us to have sex and he told us no to say anything (…) Leiner went first, and then Christian, and I was the last*”, minor, nine years old).

*Fondling/touching*: In 4.7% of cases (*n* = 32), the minor reported that the alleged offender forced the minor to touch his intimate parts. This type of act usually involved some form of violence, threat or bribes (“*he lowered my pants to do it to me, he took out his penis and asked me to touch it; he pushed me from behind to make me do it, I called out for help, he was covered my mouth and told me not to tell anybody*”, minor, seven years old). Minors usually mentioned that, after experiencing this type of act, they were subjected to anal penetration (“*he asked me to hold his buttocks, then he put his penis in my buttocks*”, minor, 10 years).

*Masturbation*: In 2.4% of cases (*n* = 16), the minor reported that the alleged offender forced the minor to masturbate him. Minors reported that this type of sexual act was perpetrated by their offenders for several months or even for years (“*my cousin locked me in and would not let me leave unless I took his penis; he did this to me four years ago, he pressed my hand against his penis and he ejaculated; he did this to me again two years ago*”, minor, 12 years old). Masturbation was frequently described as occurring together with other violent unwanted sexual acts such anal penetration or fellatio/oral intercourse.

*Minor sending photos*: In 1.2% of cases (*n* = 8), the minor reported that the alleged offender forced the minor to send sexual photos to him. Minors, most of them adolescents, reported that offenders used different social networking sites (e.g., Facebook, Whatsapp) to force them to send sexual photos (“*my 15 year old cousin started to write to me on Facebook and he started to ask me to have relations with him, I accepted (…) he continued to write to me inviting me to send him nude photos*”, minor, 12 years old). Sometimes the minors also received unwanted messages and requests for sexual photos or messages, as well as unwanted solicitation to do something sexual (“*he wrote me a lot of things, some dirty words, he wrote me ‘tomorrow you will suck my penis*”, minor, eight years old).

### 3.3. Relationship between the Type of Sexual Act and the Characteristics of the Alleged Victims and the Details of the Alleged Sexual Assaults (Objective 3)

Table 3 shows the bivariate analysis of the sexual acts the minors reported having been subjected to or forced to perform, according to their characteristics. Since the number of observations was small for some of the acts, we consolidated into the category “others” those that were less frequent. As shown in the table, very few significant relationships were observed between the type of acts the minors reported having been subjected to or forced to perform, and the characteristics of the minors. Only age and education were significantly associated with alleged assaults in which the minor reported having been anally penetrated. The Chi-Square test rejected the null hypothesis that all the minors in all age groups and education levels were equally likely to have experienced anal penetration: specifically, boys between five and nine years of age (50.7%) and between 10 and 14 years (51.2%), and boys enrolled in pre-school (50.8%) and primary school (46.9%) were more likely to have experienced this type of sexual act than those between zero and four years (36.8%) and between 15 and 17 years (35.9%), and those with no formal education (35.6%) and enrolled in secondary school (36.4%). No other significant differences were observed with respect to age and education for the other types of acts. Likewise, there were also no significant differences for all the alleged sexual acts between the four quartiles of the UNI of the municipality of residence. This implies that the frequency of prevalence of each alleged act was similar regardless of the level of social and economic development of the municipality where the minor resided.

Table 4 shows the bivariate relationship between the sexual acts the minors reported having been subjected to or forced to perform, and the details of the alleged assaults. No significant differences were observed in the prevalence of all sexual acts, according to whether the alleged assault took place on a weekday or during the weekend. Also, consistent with the findings shown in Table 3, the UNI of the municipality where the alleged assault took place was not significantly related to any type of act. Therefore, among the details of the alleged assaults, the location of the assault, the type of alleged offender, and whether money was offered, were the only variables significantly associated with the sexual acts. Alleged assaults in which the minors reported having been forced to anally penetrate the alleged offender were more likely to have occurred in public places (10.6%) than in the offender’s (4.5%) and the minor’s (2.3%) home, and in other places (4.3%). With respect to the type of alleged offender, assaults in which the minor reported having been fondled or touched by the offender were more likely to have been allegedly perpetrated by teachers (73.3%), first and second degree relatives (55.4%), and family acquaintances (45.8%); and assaults in which the minor reported having been forced to anally penetrate the alleged offender were more likely to have been perpetrated by other acquaintances (10.6%), family acquaintances (8.3%), and neighbors (7.8%). In assaults in which the minor reported having been anally penetrated, the alleged offender was less likely to have offered money to the minor (32.4%), while in assaults where the minor reported having been touched or subjected to other sexual acts, the offer of money was clearly more prevalent (51.5% and 39.7%, respectively), despite the lack of a significant relationship between the two variables. Conversely, money was more likely to have been offered in the alleged assaults where the minor reported having been forced to anally penetrate the offender, or to perform other sexual acts (17.6% and 16.2%, respectively).

## 4. Discussion

### 4.1. Main Findings

This qualitative and quantitative secondary analysis study has examined 680 cases of alleged male-on-male CSA assaults in the Colombian Caribbean Region reported to the CILMFS between the years 2017 and 2018. Analysis of the verbatim transcript of the forensic interview of each case allowed us to describe the characteristics of the alleged victims and the details of the alleged assaults; to qualitatively generate a detailed and nuanced situational narrative of sexual acts reported in each case, supported by a contextual description; and to quantitatively analyze the relationship between the type of sexual act and the characteristics of the alleged victims, as well as the details of the alleged assaults.

In relation to Objective 1, our findings revealed that the mean age of the minors was approximately eight years, which is line with previous research showing a higher prevalence of CSA in the ages between eight and 12 years, and a lower prevalence at younger and more adolescent ages [56,57,58]. The results were also in line with the findings of a considerable number of studies showing that perpetrators of CSA tend to be individuals who are known to either the victim or the family, both in Western [56,59,60] and non-Western countries [23,61,62,63,64,65,66]. In our study, almost fifty per cent of the alleged offenders were relatives or acquaintances of the family, a percentage that is similar to that reported in the study by Speizer et al. [61] in Honduras, El Salvador, and Guatemala. Furthermore, only less than ten per cent of the alleged offenders were strangers to the minor, a frequency which is consistent with the findings of Pineda-Lucatero et al. [25] in Mexico. In contrast with the results of previous studies reporting that assaults usually take place during the weekend, this study showed that most of the alleged assaults occurred during weekdays [67,68]. Finally, another remarkable finding is that, in most of the cases, the municipality of residence of the minor, as well as the municipality where the alleged assault took place, were in the quartile of municipalities with the lowest percentage of people with their basic needs unsatisfied. While this finding is in line with the results of other studies that show no association between a high prevalence of CSA and low family income [69,70,71], we should not necessarily conclude that such an association does not exist. As shown in the study by Ramirez et al. [32], in Colombia strong ‘machismo’ values are highly correlated with a lower family income. Therefore, our findings might result from a tendency among low income families to underreport cases of sexual abuse in order to avoid calling into question values of hegemonic masculinity that they uphold.

With respect to Objective 2, the forensic interviews allowed the minors or their relatives to speak openly using their own words, and in a comfortable and relaxed way, about the specific incidents that occurred during the alleged assaults they reported. Since the descriptions of the alleged assaults were open-ended and qualitative in nature, we were able to carry out a fine-grained and contextualized analysis of the sexual acts that the minors reported having been subjected to or forced to perform. Our findings showed that male children and adolescents reported a wide range of sexual acts, with anal penetration (with the penis, finger, or other objects) being the most frequent, followed by fondling and touching, and oral sex. Previous studies have also reported anal penetration and forcible fondling as the most prevalent sexual acts occurring in sexual assaults perpetrated by males [72,73]. In our findings, a common pattern in most of the alleged acts was the high level of violence involved, including violent force, psychological coercion, threats, and, in some cases, physical injuries. According to several authors [25], sexual assaults reported by male children and adolescents tend to differ greatly from those reported by females, as they involve higher levels of physical violence. While the male-only composition of our sample did not allow us to test this hypothesis, our findings provide strong evidence of the physically violent nature of the sexual acts that the male minors in our sample reported having been subjected to or forced to perform.

In relation to Objective 3, we observed few significant relationships between the types of alleged sexual acts identified in the forensic interviews and the characteristics of the alleged victims as well as the details of the alleged assaults. Only incidents in which minors reported having been anally penetrated or forced to anally penetrate the alleged offender showed several significant differences for some of the independent variables. Specifically, findings revealed that having been subjected to penile-anal penetration was more likely to occur among boys of ages five to 14 and among those enrolled in preschool, while having been forced to perform penile-anal penetration on the alleged offender was more likely to take place in public spaces, and the alleged perpetrators tended to be acquaintances and neighbors. Also, these two types of anal assaults showed a significant relationship with the offer of money by the alleged offender: in cases when the child reported having been anally penetrated by the alleged offender, money was less likely to be offered, while in cases when the child reported having been forced to anally penetrate the alleged offender, money was more likely to be offered. In such cases, the alleged offender might anticipate potential problems in completing the assault (which would not be the case when he plays the active role), and this circumstance might lead the alleged offender to offer money to the minor. The findings related to Objective 3 should be considered only exploratory as, to our best knowledge, no other studies have previously examined the personal and contextual factors surrounding each type of sexual act. While these results provide interesting insights that can contribute to advancing our knowledge on the nature and characteristics of sexual acts occurring in male-on-male alleged CSA, they need to be confirmed and refined in further quantitative and qualitative studies.

### 4.2. Strengths and Limitations

To our knowledge, this is the first study that has examined incidents of alleged male-on-male CSA in Colombia. In light of the scarcity of data on CSA in South American countries [20,74], the study also fills an important gap in the knowledge and understanding of sexual abuse among minors in this geographical area. Another strength is that the data used in the study was generated from forensic interviews, which were a non-threatening environment in which minors were able to provide a detailed description of the alleged sexual assault.

The study is also subject to a few limitations. First, only alleged offenses reported to the CILMFS were examined. Therefore, the sample is not likely to be representative of all the cases of CSA in the region, particularly because of the high prevalence of underreporting. Second, since the study is based on a secondary analysis of archived data, the findings are subjected to limitations typical to this type of analysis [35]. In particular, since we did not collect the data, we did not have detailed information on the characteristics of the alleged offender or the opportunity to use standardized instruments to assess the truthfulness of the allegations or to collect further information about the number and frequency of alleged CSA incidents experienced by the minor. Third, forensic interviews may also be subject to recall bias caused by systematic errors that occur when the minor does not accurately remember the incident or omits some of its details. Fourth, as argued by Lipian, Mills, and Brantman [75], children’s testimonies before and during the forensic interviews may have been contaminated by the caregivers and family members’ own beliefs, perceptions, and misinterpretations of the incident.

### 4.3. Implications for Policy and Practice

Our findings may have some implications for the development of interventions to assist victims and for implementing preventive actions. Teachers, parents, and other care providers may need training to be able to recognize symptoms of and risk factors for CSA. Additionally, forensic professionals may need specific training to carry out forensic interviews with young children and to interpret their testimonies, taking into consideration their stage of development. This is particularly in light of our findings on the prevalence of reported sexual assaults on children of a very young age. While social agencies frequently develop programs to address the harm to children caused by abuse, there is a need for action to prevent CSA before it occurs. A public health approach is needed that can address CSA at a micro and macro societal levels by proposing evidence-based and culturally preventative actions [76,77]. These would include government-led interventions to facilitate the arrest and prosecution of offenders in order to protect the young people. Also, schools and the community need to provide adequate information about children’s rights and gender equality in order to help children avoid sexual assaults and report any incidents to the authorities. These actions are especially important in Colombia, where “machismo” and cultural attitudes related to hegemonic masculinity are prevalent [32]. Traditional gender roles and stereotypes, which depict men as dominant, unemotional, heterosexual and physically though, are quite firmly established in Colombia and all other Latin American countries [78,79]. Furthermore, schools and the community should provide training for parents to help their children recognize abusive situations and to be able to take the necessary actions to keep themselves safe. Since our findings show that children reported having been frequently abused by alleged perpetrators close to them, children need to be trained to be able to recognize appropriate and inappropriate forms of contact.

### 4.4. Considerations for Future Research

Further research is needed to gather more detailed information on the characteristics of offenders such as their age, municipality of residence, and socioeconomic status. Also, additional information about offenders should be collected, such as the psychosocial motives that might lead them to offend or the strategies used to prevent victims from disclosing or reporting the assault. Furthermore, a better contextual understanding is needed of the influence on the prevalence of CSA of factors such as political and economic circumstances (i.e., poverty, armed conflict) and social and cultural values (i.e., gender roles and gender socialization).

## 5. Conclusions

Male-on-male CSA in Colombia is a prevalent phenomenon despite being little known and infrequently described in research. Masculinist cultural attitudes often prevent children and their families from reporting sexual assaults to the authorities. According to this study, male-on-male CSA allegedly occurs mostly with young victims and is allegedly perpetuated by offenders who are close to the minors or their families, using high levels of violence. Training-based programs for teachers and parents, along with other public health initiatives, are likely needed in order to address this type of CSA. Further research is required to gain a better contextual understanding of the cultural and social nature of this phenomenon in the Caribbean Latin American countries.

## Figures and Tables

**Table 1:** Characteristics of the alleged victims (*N* = 680).

	Total
Age, Mean (Standard Deviation)	8.66 (4.08)
Education, *n* (%)	
No formal Education	101 (14.9)
Preschool	378 (55.6)
Primary School	175 (25.7)
Secondary School	22 (3.2)
Unsatisfied Needs Index of the Municipality of Residence (in Quartiles), *n* (%)	
Quartile 1	498 (73.5)
Quartile 2	137 (20.2)
Quartile 3	26 (3.8)
Quartile 4	17 (2.5)

**Table 2:** Details of the alleged assaults (*N* = 680).

	Total
Day of the Week, *n* (%)	
Weekday	526 (77.4)
Weekend	154 (22.6)
Location, *n* (%)	
Public/Outdoors/Park	123 (18.1)
Alleged offender’s home	201 (29.6)
Minor’s home	217 (31.9)
Other	139 (20.4)
Unsatisfied Needs Index of the Municipality where the Alleged Assault Took Place (in Quartiles), *n* (%)	
Quartile 1	490 (72.1)
Quartile 2	145 (21.3)
Quartile 3	28 (4.1)
Quartile 4	17 (2.5)
Type of Alleged Offender, *n* (%)	
First and second degree relative	101 (14.9)
Third degree relative	184 (27.1)
Family acquaintance	24 (3.5)
Friend of the minor	58 (8.5)
Neighbor	129 (19.0)
Teacher	15 (2.2)
Other acquaintance	113 (16.6)
Stranger	56 (8.2)
Alleged Offender offers Money, *n* (%)	
No	611 (90.0)
Yes	68 (10.0)

**Table 3:** Sexual acts the minor reported having been subjected to or forced to perform, by the characteristics of the alleged victims (*N* = 680).

	Sexual Acts Minor Reported Having Been Subjected to ^1^			Sexual Acts Minor Reported Having Been Forced to Perform ^1^		
	Anal Penetration (Penis)	Fondling/Touching	Other	Fellatio/Oral Intercourse	Anal Penetration (Penis)	Other
Age (%)						
0–4	36.8	40.2	32.5	14.5	6.8	8.5
5–9	50.7	40.7	34.1	15.5	3.1	10.0
10–14	51.2	39.2	36.4	13.9	6.2	5.3
15–17	35.9	51.6	40.6	12.5	4.7	14.1
*X*^2^ (df = 3)	11.137 ^a^	3.253	1.473	0.551	3.770	6.060
Cramer’s V ^2^	0.128	-	-	-	-	-
Education (%)						
No formal Education	35.6	42.6	39.6	16.8	7.9	5.0
Preschool	50.8	40.2	33.3	15.1	3.2	10.3
Primary School	46.9	40.0	34.3	14.3	6.3	6.9
Secondary School	36.4	59.1	54.5	0.0	9.1	13.6
*X*^2^ (df = 3)	8.412 ^a^	3.242	5.110	4.236	5.964	4.442
Cramer’s V	0.112	-	-	-	-	-
Unsatisfied Needs Index of the Municipality of Residence (in Quartiles) (%)						
Quartile 1	47.4	42.4	36.9	15.9	5.8	8.4
Quartile 2	46.0	39.4	29.9	9.5	1.5	8.8
Quartile 3	46.2	30.8	30.8	11.5	7.7	11.5
Quartile 4	41.2	41.2	35.3	23.5	0.0	11.8
*X*^2^ (df = 3)	0.323	1.625	2.558	4.790	5.736	0.510
Cramer’s V	-	-	-	-	-	-
Total (%)	47.1	41.2	35.1	14.6	4.9	8.7

^a^ Significant at *p* ≤ 0.05. ^1^ Categories are not mutually exclusive. ^2^ Cramer’s V measures the strength of the relationship between two categorical variables. Cramer’s V values range from 0 to 1, with values closer to 1 indicating a stronger relationship.

**Table 4:** Sexual acts the minor reported having been subjected to or forced to perform, by the details of the alleged assaults (*N* = 680).

	**Sexual Acts Minor Reported Having Been Subjected to ^1^**			**Sexual Acts Minor Reported Having Been Forced to Perform ^1^**		
	**Anal Penetration (Penis)**	**Fondling/Touching**	**Other**	**Fellatio/Oral Intercourse**	**Anal Penetration (Penis)**	**Other**
Day of the week (%)						
Weekday	45.6	41.6	35.7	15.2	4.8	8.9
Weekend	51.9	39.6	33.1	12.3	5.2	7.8
*X*^2^ (df = 1)	1.910	0.202	0.360	0.790	0.050	0.196
Cramer’s V	-	-	-	-	-	-
Location (%)						
Public/Outdoors/Park	40.7	39.8	31.7	13.8	10.6	6.5
Alleged offender’s home	46.3	43.8	33.3	18.4	4.5	8.0
Minor’s home	49.8	39.6	37.8	13.8	2.3	11.5
Other	49.6	41.0	36.7	10.8	4.3	7.2
*X*^2^ (df = 3)	3.090	0.870	1.738	4.128	11.905 ^b^	3.464
Cramer’s V	-	-	-	-	0.132	-
Unsatisfied Needs Index of the Municipality where the Alleged Assault Took Place (in Quartiles) (%)						
Quartile 1	47.6	42.2	37.1	16.3	5.7	9.0
Quartile 2	46.9	39.3	28.3	8.3	1.4	6.9
Quartile 3	42.9	32.1	35.7	10.7	10.7	10.7
Quartile 4	41.2	41.2	35.5	23.5	0.0	11.8
*X*^2^ (df = 3)	0.484	1.383	3.864	7.265	7.527	0.988
Cramer’s V	-	-	-	-	-	-
Type of Alleged Offender (%)						
First and second degree relative Third degree relative Family acquaintance Friend of the minor Neighbor Teacher Other acquaintance Stranger *X*^2^ (df = 7) Cramer’s V	43.6 54.9 41.7 50.0 44.2 26.7 42.5 48.2 9.420 -	55.4 39.1 45.8 25.9 37.2 73.3 39.8 39.3 22.049 ^b^ 0.180	38.6 34.2 41.7 24.1 36.4 33.3 40.7 26.8 7.497 -	7.9 18.5 12.5 17.2 19.4 20.0 10.6 7.1 12.920 -	0.0 1.6 8.3 5.2 7.8 0.0 10.6 5.4 21.214 ^b^ -	10.9 7.6 12.5 5.2 7.0 6.7 10.6 10.7 3.610 -
Alleged Offender offers Money (%) No Yes *X*^2^ (df = 1) Cramer’s V	48.8 32.4 6.621 ^b^ 0.099	40.1 51.5 3.266 -	34.7 39.7 0.673 -	14.1 17.6 0.632 -	3.4 17.6 26.722 ^c^ 0.198	7.9 16.2 5.339 ^a^ 0.089
Total *(%)*	47.1	41.2	35.1	14.6	4.9	8.7

^a^ Significant at *p* ≤ 0.05; ^b^ Significant at *p* ≤ 0.01; ^c^ Significant at *p* ≤ 0.001. ^1^ Categories are not mutually exclusive.
